# Growth and Stress-induced Transformation of Zinc blende AlN Layers in Al-AlN-TiN Multilayers

**DOI:** 10.1038/srep18554

**Published:** 2015-12-18

**Authors:** Nan Li, Satyesh K. Yadav, Jian Wang, Xiang-Yang Liu, Amit Misra

**Affiliations:** 1 Materials Physics and Applications Division, MPA-CINT, Los Alamos National Laboratory, Los Alamos, New Mexico 87545, USA; 2 Materials Science and Technology Division, MST-8, Los Alamos National Laboratory, Los Alamos, New Mexico 87545, USA; 3 Department of Mechanical and Materials Engineering, University of Nebraska-Lincoln, Lincoln, NE 68588, USA; 4 Department of Materials Science and Engineering, University of Michigan, Ann Arbor, Michigan 48109, USA

## Abstract

AlN nanolayers in sputter deposited {111}Al/AlN/TiN multilayers exhibit the metastable zinc-blende-structure (z-AlN). Based on density function theory calculations, the growth of the z-AlN is ascribed to the kinetically and energetically favored nitridation of the deposited aluminium layer. *In situ* nanoindentation of the as-deposited {111}Al/AlN/TiN multilayers in a high-resolution transmission electron microscope revealed the z-AlN to wurzite AlN phase transformation through collective glide of Shockley partial dislocations on every two {111} planes of the z-AlN.

AlN is characterized by high ionicity, short bond length, low compressibility, high thermal conductivity, and a wide band gap^1^,^2^,^3^,^4^. These properties make it useful in many applications such as electronic substrates, heat sinks, electronic packaging, and high-temperature transistors. Different techniques such as chemical vapor deposition, molecular beam epitaxy, pulsed laser deposition and reactive magnetron sputtering have been used to deposit AlN films^5^,^6^. The equilibrium structure of AlN at ambient temperature and pressure is hexagonal wurtzite (w-AlN)^5^,^6^. Additionally, AlN also exists as a metastable cubic zinc-blende structure (z-AlN) or the high-pressure cubic rock-salt variant (r-AlN), as predicted using density functional theory^1^,^2^,^3^,^4^. There has been a growing interest in the metastable cubic films of AlN recently to achieve novel and enhanced mechanical and functional properties that are not observed in the hexagonal structure.

By tailoring substrate in terms of crystal structure, substrate orientation, and elastic mismatch, the cubic crystal structure of AlN layer can be grown in superlattice systems and the stability of the cubic structures strongly depends on its layer thickness^7^,^8^,^9^,^10^,^11^,^12^,^13^,^14^. The high-pressure rock-salt AlN (r-AlN) was stabilized in AlN/TiN(001) superlattices with AlN layer thickness less than 2.0 nm^10^, and in AlN/VN (001) superlattices with AlN layer thickness less than 4.0 nm due to the smaller lattice mismatch between r-AlN and VN^11^,^12^. Epitaxial metastable zinc-blende-structure z-AlN layer was synthesized in Al(001)-TiN(001) superlattices by ultrahigh vacuum magnetron sputter deposition^13^. At an annealing temperature T = 600 °C, z-AlN was formed by the solid-state reaction according to the interaction 4Al + TiN→Al_3_Ti + AlN and pseudomorphically grown between cubic TiN and tetragonal Al_3_Ti layers^13^. The z-AlN was also stabilized in AlN(001)/W(001) superlattices as the layer thickness less 1.5 nm because of the smaller interfacial energy of z-AlN/W interface than r-AlN/W or w-AlN/W interface^14^. With increasing the thickness of AlN layers, a selected-area electron diffraction (SAED) pattern or a high angle x-ray diffraction scan indicates the rock-salt to hexagonal transformation^10^,^11^,^12^ and the zinc blende to hexagonal transformation^13^,^14^, because the superlattices become energetically unfavorable or layer roughening results in a loss of coherent interfaces^13^. However, the transformation path has not been characterized in a transmission electron microscope (TEM).

In this Letter, we synthesized the metastable z-AlN nanolayers between Al(111) and TiN(111) layers during physical vapor deposition and addressed the growth mechanisms based on first principle density function theory calculations. Through *in situ* indentation in a high-resolution TEM we further characterized the z-AlN to w-AlN transformation path with collective glide of Shockley partial dislocations on every two hexagonal close packed planes.

## Results

Thin multilayers composed of alternating Al–AlN–TiN individual layers were deposited at room temperature using reactive direct current (dc) magnetron sputtering on Si substrates (with a top layer of amorphous SiO_2_)^15^,^16^. The deposition was performed onto silicon (100) substrate at room temperature in a balanced dc magnetron sputtering system using high-purity (99.999%) aluminium and titanium targets, operated at 100 W DC cathode power and 6.5 cm away from the substrate. The vacuum pumping system consisted of a turbomolecular pump backed by a mechanical pump which provided a base pressure lower than 10^−6^ mbar; the working pressure was 2 ~ 4 × 10^−3^ mbar. Argon and nitrogen gases were introduced into the chamber by separated mass flow controllers. The argon flow was 25 sccm during multilayer deposition. Before the deposition of Ti, the nitrogen flow was set at 15 sccm and 300V DC bias was applied for 10 s. Afterwards, the deposition of Ti starts and both nitrogen flow and Ti deposition will be turned off before ensuing Al deposition with the process pressure remaining the same at 6 × 10^−3^ torr.

**Figure 1:**
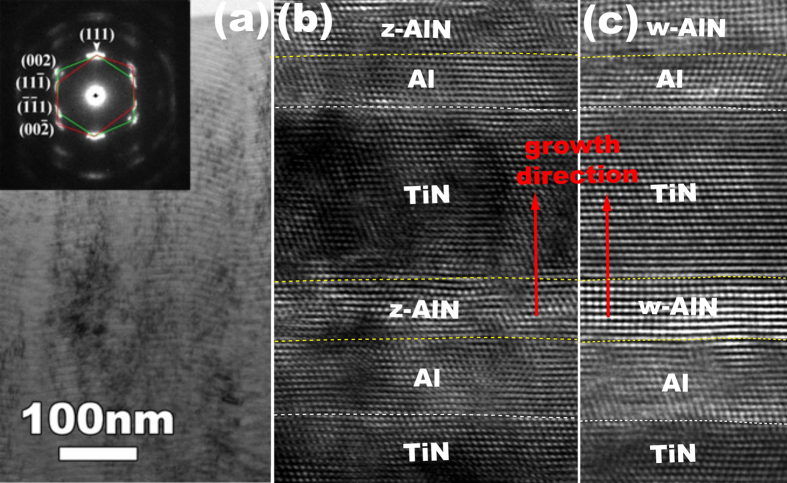
(**a**) TEM image of the as-deposited films with the individual layer thickness 5 nm. (**b**) A magnified HRTEM image of the as-deposited films showing crystal structures of Al-AlN-TiN trilayers. (**c**) A magnified HRTEM image of the deformed film showing crystal structures of Al-AlN-TiN trilayers. The white dashed lines indicate the interface between the TiN and Al. The yellow dashed lines indicate the Al and AlN interface and the AlN and TiN interface.

Figure 1 shows cross-sectional TEM images of the Al-AlN-TiN multilayers with columnar structure. The diffraction pattern (DP) of the as-deposited films with the individual layer thickness of 3.5 nm Al, 1.5 nm AlN, and 5 nm TiN and the high resolution TEM image in Fig. 1b confirm that Al, AlN and TiN have face centered cubic structures (fcc), the orientation relation: (111)_Al_||(111)_AlN_||(111)_TiN_||interface and <110>_Al_||<110>_AlN_||<110>_TiN_, the growth direction along [111]. The DP also shows the twin orientation which corresponds to a relative rotation of 60° between adjoining columns around <111> growth direction^15^. After indentation testing, a hexagonal close packed (hcp) structure AlN (hcp-AlN or w-AlN), corresponding to the original fcc-AlN or z-AlN layer, is characterized in-between the Al and TiN layers as shown in Fig. 1c. The most intriguing findings are the growth of metastable z-AlN layers only on top of Al layer and the z-AlN to w-AlN transformation after mechanical loading. However, two open issues are consequently raised: the formation mechanism of z-AlN layer and the transformation path.

**Figure 2:**
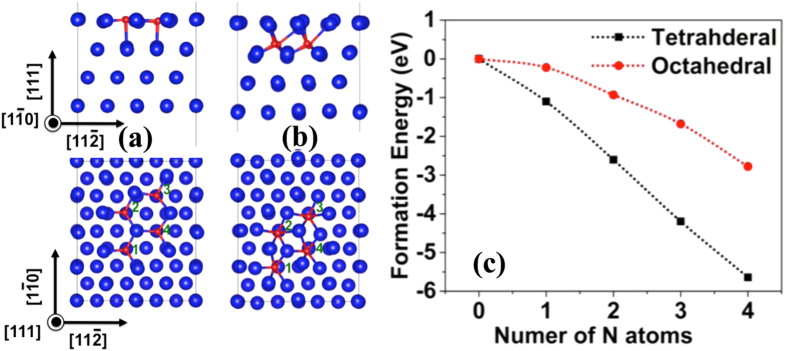
Atomic structure of Al slabs indicating four positions of (**a**) tetrahedral and (**b**) octahedral.(**c**) Formation energy as a function of the number of N atoms.

Taking the deposition process into account, the nitrogen flow was set at 15 sccm before the deposition of Ti, but turned off before depositing Al. Al layer will grow in a fcc structure on the TiN layer before depositing the next TiN layer. We thus proposed that the z-AlN was formed by direct nitridation of the deposited fcc-Al layer. Correspondingly, AlN layer does not grow on the TiN layer because the nitrogen flow is turned off. To examine this proposed growth mechanism of z-AlN layer, we performed first principles density function theory (DFT) calculations (the technical details can be found in Supplementary)^17^,^18^. Starting with a fcc Al (64 atoms), the nitridation of the deposited Al layer can be realized in two ways. A nitrogen atom can occupy the tetrahedral site or octahedral interstitial site in a fcc structure, corresponding to the formation of z-AlN or r-AlN respectively, as shown in Fig. 2a,b (also see Fig. 5a,b). DFT calculations are performed by adding N atoms one by one into the tetrahedral sites or octahedral sites in the fcc-Al. Figure 2c shows the variation of the formation energy with the number of the added N atoms with respect to the occupation of tetrahedral sites or octahedral sites. The result suggests that the nitridation of the Al layer in tetrahedral sites is energetically preferred, i.e, the formation of the z-AlN is kinetically and energetically favored compared to the r-AlN. In addition, it is worthy of mentioning that both w-AlN(0001)-Al(111) and z-AlN(111)-Al(111) interfaces are subjected to the same lattice mismatch where the shortest bond length is 0.286 nm in Al, 3.11 nm in w-AlN and 3.11 nm in z-AlN^1^,^2^,^3^,^4^,^5^. However, the z-AlN is metastable phase with a higher cohesive energy than the stable w-AlN phase and the z-AlN will transform into w-AlN from thermodynamic point of view.

**Figure 3:**
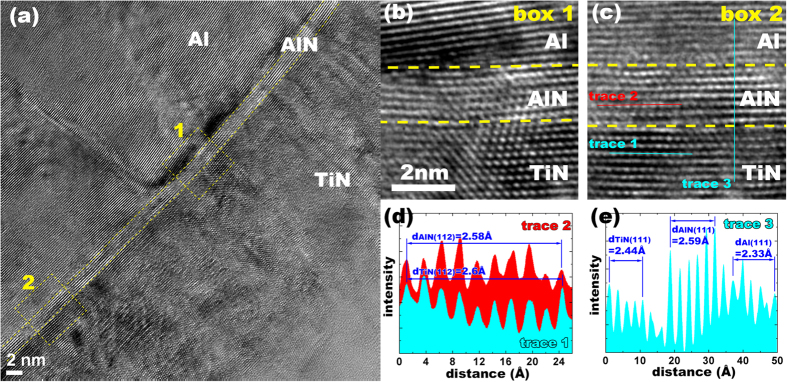
(**a**) TEM image of the as-deposited Al-AlN-TiN trilayer. The AlN layer has the zinc blende structure, as be magnified in (**b**–**e**) the measurements of lattice parameters in the TiN, z-AlN and Al layers.

**Figure 4:**
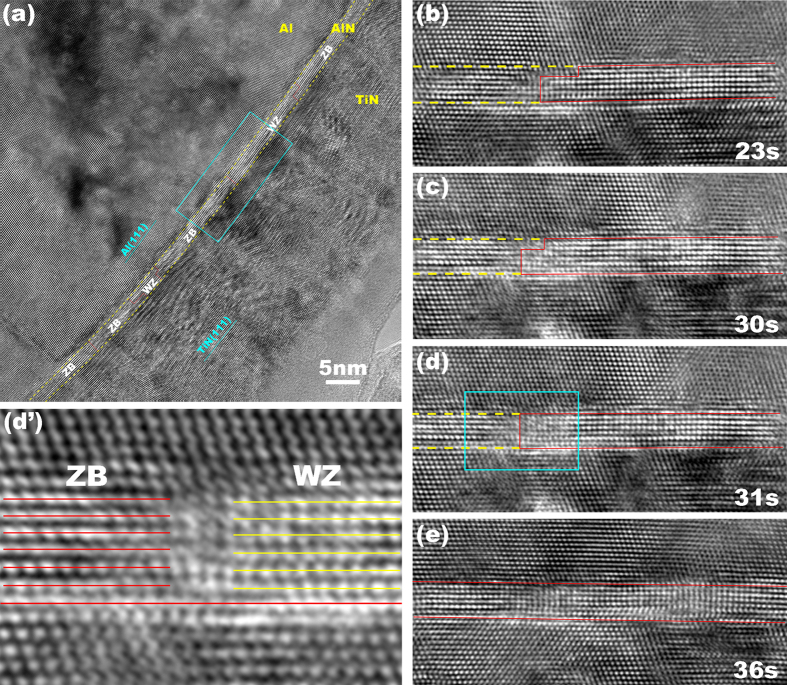
Phase transformation in the AlN layer. HRTEM images are from *in situ* indentation test. (**a**) One HRTEM image shows the heterogeneous phase transformation in the AlN layer. WZ and ZB indicate the w-AlN and z-AlN phase. (**b**–**e**) HRTEM images of four snapshots of the region outlined in the blue rectangle in (**a**), showing the w-AlN nucleated and propagate towards the right direction. A sharp interface between the w-AlN and z-AlN was observed in all images and magnified in (**d**’).

To explore the transformation path, we deposited the Al-AlN-TiN trilayer with the same deposition parameters (Fig. 3). Before indentation testing, we characterized the as-deposited film using HRTEM. The AlN layer is about 1.5 nm thick and adopts the zinc blende structure (z-AlN). Figure 3b,c show HRTEM images of the z-AlN and Fig. 3d,e characterized the distance of atom columns along the <112> direction, 0.258 nm, and the interplanar spacing of the (111) plane in the z-AlN, 0.259 nm. These are consistent with the crystallographic parameters of a z-AlN, 0.267 nm along the <112> and 0.252 nm along the <111> direction^1^,^2^,^3^. After the indentation testing, the z-AlN gradually transforms into the w-AlN, as shown in Fig. 4. We further characterized the interplanar spacing of the (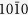) plane in the w-AlN (0.270 nm), and that of the (0001) plane (0.247 nm), as shown in Figure S1 in the Supplementary. These are consistent with the crystallographic parameters of a w-AlN, 0.269 nm along the 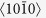
and 0.249 nm along the <111> direction^1^,^2^,^3^.

Phase transformation processes are revealed with several high-resolution transmission electron microscope (HRTEM) images of the Al-AlN-TiN trilayer during *in situ* indentation testing (see Movie I in the Supplementary)^15^,^19^. Figure 4b–e show several TEM images of the AlN layer with respect to the indentation time. w-AlN with the height of six (0001) atomic layers forms and propagates towards the left in Fig. 4b–d. A sharp interface between the z-AlN and w-AlN forms in the AlN layer as shown in Fig. 4d and magnified in Fig. 4d’. Finally, the z-AlN layer transforms entirely into the w-AlN layer (Fig. 4e) and the corresponding layer thickness decreases ~4.6%.

## Discussion

Two microstructural features are worthy of further discussion, i.e., the interface between the z-AlN and w-AlN crystals is sharp and the layer thickness remains unchanged after the phase transformation. These two features can be well addressed based on the collective gliding of three Shockley partials on every two (111) planes^20^ (Figure S2 in the Supplementary). Starting with a fcc structure, a hcp structure can be created by the glide of any of the three Shockley partial dislocations **b**_**1**_, **b**_**2**_, **b**_**3**_on every two (111) planes^20^. **b**_**1**_, **b**_**2**_, and **b**_**3**_are equal to 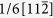, 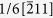 and 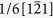 on the (111) plane, respectively. Through the glide of a set of partial dislocations with a repeatable sequence **b**_**2**_:**b**_**1**_:**b**_**3**_ on every two {111} plane in a fcc structure, a six-atomic layer hcp structure can be created. It is worthy of mentioning that the three Shockley partials have the net zero Burgers vector^21^,^22^,^23^, attract each other and form the sharp interface between the z-AlN and w-AlN, as demonstrated by atomistic simulation in Figure S3 in the Supplementary.

**Figure 5:**
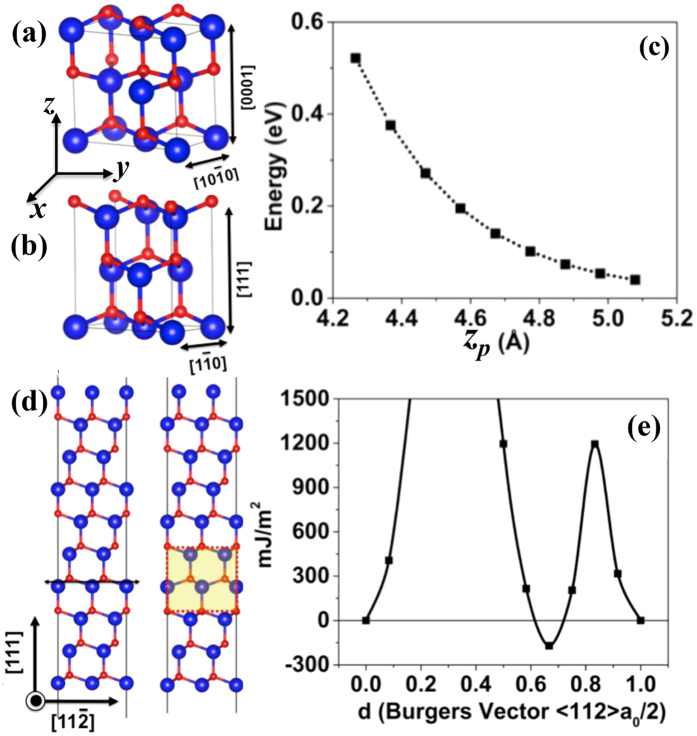
Super-cell of (**a**) wurtzite and (**b**) zinc blende used for modeling uniaxial compression. (**c**) Energy per a pair of atoms AlN as a function of strain under uniaxial compression. (**d**) Atomic structures of (the left) supercell used to model the generalized stacking fault energy in AlN and (the right) the local wurtzite AlN by displacing two layers in the z-AlN. (**e**) The GSF energies as a function of shear displacement along <112> on {111} plane.

Once the z-AlN is subjected to the normal compression, *in situ* TEM observation revealed the transformation of the z-AlN to the w-AlN. Figure 5c shows the difference in the bulk energy of the z-AlN and w-AlN, 
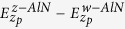
, as a function of the unit height of the <111> in the z-AlN and the <0001> in the w-AlN. The energy difference increases with decreasing the unit height corresponding to uniaxial compression along the growth direction, indicating that the z-AlN to the w-AlN transformation is energetically preferred. Although the w-AlN has a lower bulk energy than the z-AlN, the formation of the w-AlN may not occur during deposition because the z-AlN to the w-AlN transformation must overcome kinetic energy barriers corresponding to the shear along one of <112> directions on (111) plane. Figure 5e shows the generalized stacking fault energy profile of the (111) plane in the z-AlN and the corresponding energy barrier of 1200 mJ/m^2^. As a result of shearing the z-AlN along 1/6<112>, a two-layer stacking fault created in the z-AlN corresponds to a local w-AlN phase embedded in the z-AlN crystal (Fig. 5d) which lowers the bulk energy of the z-AlN, suggesting that the z-AlN layer is kinetically stabilized.

In summary, we synthesized the metastable z-AlN in Al-TiN multilayers using magnetic sputtering technique. Combining DFT calculations and *in situ* nanointdentation testing in a TEM, the growth of the z-AlN is ascribed to the kinetically favored nitridation of the deposited Al layer and the z-AlN layer is kinetically stabilized. Under mechanical loading, the structural transformation from the z-AlN to the w-AlN is accomplished via collective glide of Shockley partial dislocations on {111} planes of the z-AlN, resulting in a heterogeneous nanolayer containing both z-AlN and w-AlN in-between the Al and TiN layers. The finding from this work opens a window to fabricate z-AlN in multilayers. The future work will focus on the synthesis of Al/z-AlN multilayers that could have multifunctional property of high strength and piezoelectric materials^24^,^25^, because the ability of metal-ceramics multilayer to stand high strain can be used to harness piezoelectric properties of AlN.

## Methods

### Characterization and Nanoindentation

The film thickness of all samples is around 2 μm. TEM samples were prepared by mechanical polishing to a final thickness of 20–30 μm with diamond lapping film, followed by ion-milling using a Gatan™ PIPS^®^ instrument operating at 3–5 kV. Indentation experiments were conducted inside a FEI Tecnai F30 field emission gun transmission electron microscopy equipped with a Nanofactory TEM-STM system. The TEM was operated at 300 kV, with a point-to-point resolution around 0.2 nm. The TEM foils were attached to a piezo-operated scanning tunneling microscope (STM) probe with silver paint, which served as one end of a Nanofactory TEM-STM platform. An etched W tip was the other end of platform. The STM probe with the W tip was compressed onto the TEM foil with the indentation direction perpendicular to the interface plane. The loading rate is controlled to be 1 nm/s. We recorded the videos during indentation by a CCD (charge-coupled device) camera at 3 frames per second.

### Density Function Theory Calculations

The DFT calculations have been performed using the Vienna Ab initio Simulation Package (VASP)^26^, in which the Perdew, Burke, and Ernzerhof (PBE)^27^ generalized gradient approximation (GGA) exchange-correlation functional and the projector-augmented wave (PAW) method^27^ have been employed. The valence configuration for Al is [Ne]3**s**^2^3**p**^1^ with cutoff radius 1.40 Å, and N is [He]2**s**^2^2**p**^3^ with cutoff radius 0.74 Å. For all the DFT calculations, a plane wave cutoff of 500 eV for the plane wave expansion of the wave functions is used to obtain highly accurate forces. Force tolerance for the structural relaxation is 0.05 eV/Å. The calculated lattice parameters of AlN in various phases are in excellent agreement with experiments. For the w-AlN, the lattice constant is equal to 0.312 nm comparable to the experiment 0.311 nm^28^; for the z-AlN, the lattice constant is equal to 0.440 nm comparable to the experiment 0.437 nm^29^, and for the r-AlN, the lattice constant is equal to 0.407 nm comparable to the experiment 0.406 nm^1^.

In order to explain the formation of zinc blende phase over rock salt phase of AlN by adding N atoms in Al crystal, we calculate formation energy of AlN_(1−x)_ as Al is exposed to N. Each of N atoms then is filled sequentially in the Al slab constituted of 64 atoms. We define the formation energy as 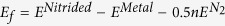, where 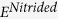 is the energy of the metal slab and *n* nitrogen atoms, 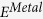 is the energy of the metal slab (64 Al atoms) and 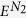 is the energy of N_2_.

## Additional Information

**How to cite this article**: Li, N. *et al.* Growth and Stress-induced Transformation of Zinc blende AlN Layers in Al-AlN-TiN Multilayers. *Sci. Rep.* **5**, 18554; doi: 10.1038/srep18554 (2015).

## Supplementary Material

Supplementary Information

Supplementary Movie 1
