# Supplementary material for: Growth and Stress-induced Transformation of Zinc blende AlN Layers in Al-AlN-TiN Multilayers
Source: Sci Rep. 2015 Dec 18;5:18554. doi: 10.1038/srep18554 (PMC4683522; doi:10.1038/srep18554)
Supplement: Supplementary Movie 1 [file srep18554-s2.pdf]

Supplementary Information

Growth and Stress-induced Transformation of Zinc blende AlN Layers in Al-AlN-TiN Multilayers

Nan Li[1], Satyesh K. Yadav[2], Jian Wang[3,*], Xiang-Yang Liu[2], Amit Misra[4]

[1] Materials Physics and Applications Division, MPA-CINT, Los Alamos National Laboratory, Los Alamos, New Mexico 87545, USA
[2] Materials Science and Technology Division, MST-8, Los Alamos National Laboratory, Los Alamos, New Mexico 87545, USA
[3] Department of Mechanical and Materials Engineering, University of Nebraska-Lincoln, Lincoln, NE 68588, USA
[4] Department of Materials Science and Engineering, University of Michigan, Ann Arbor, Michigan 48109, USA

Figure S1. TEM image of the deformed Al-AlN-TiN trilayer.

Figure S2. Schematic of phase transformation path.

Figure S3. DFT calculation of the zinc blende and wurtzite interface.

* Corresponding author: Corresponding author: Dr. Jian Wang, Phone: +1 402-472-2375, E-mail: jianwang@unl.edu; Dr. Nan Li, Phone: +1 505-665-1857, E-mail: nanli@lanl.gov

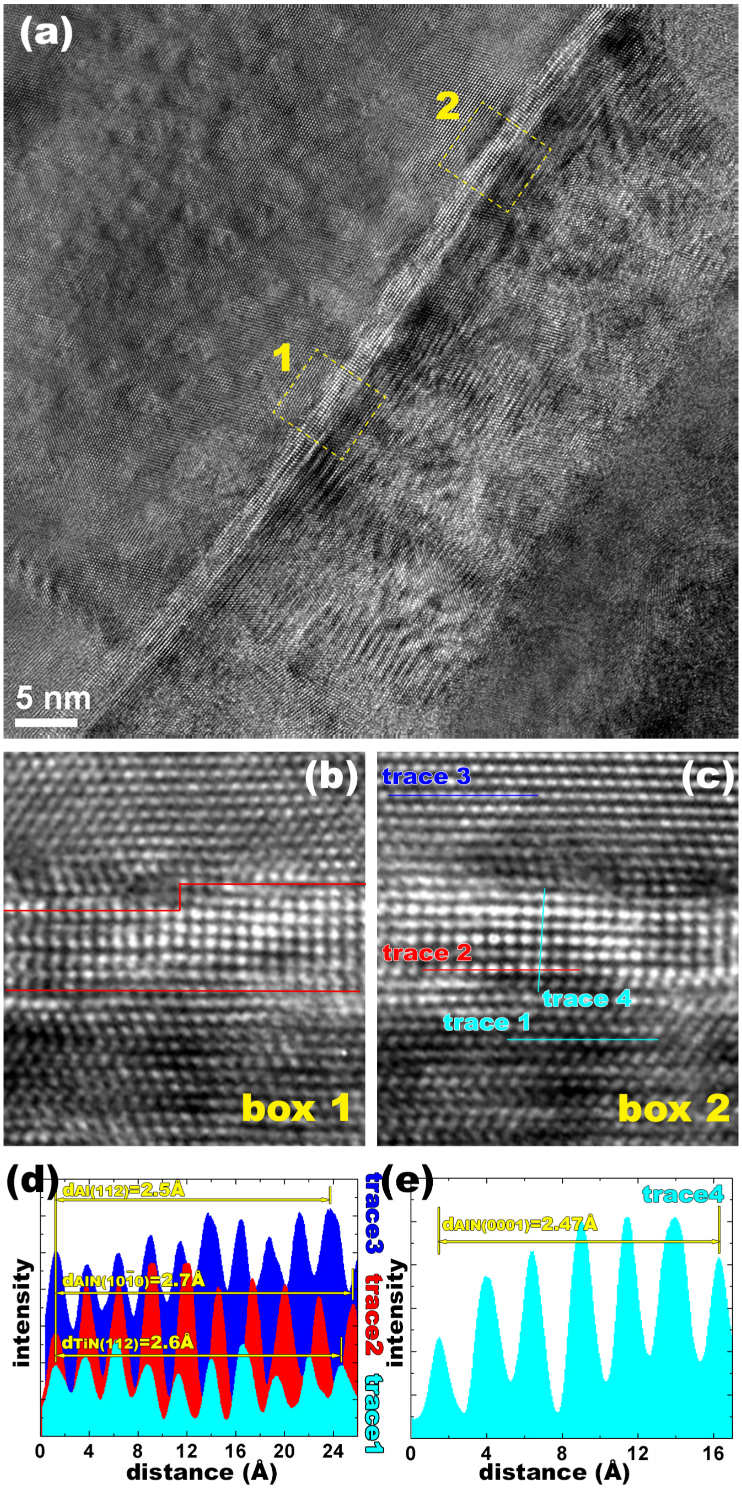

Figure S1. (a) TEM image of the deformed Al-AlN-TiN trilayer. The AlN layer has the wurzite structure, as be magnified in (b) and (c). (d) and (e) the measurements of lattice parameters in TiN, w-AlN and Al layers.

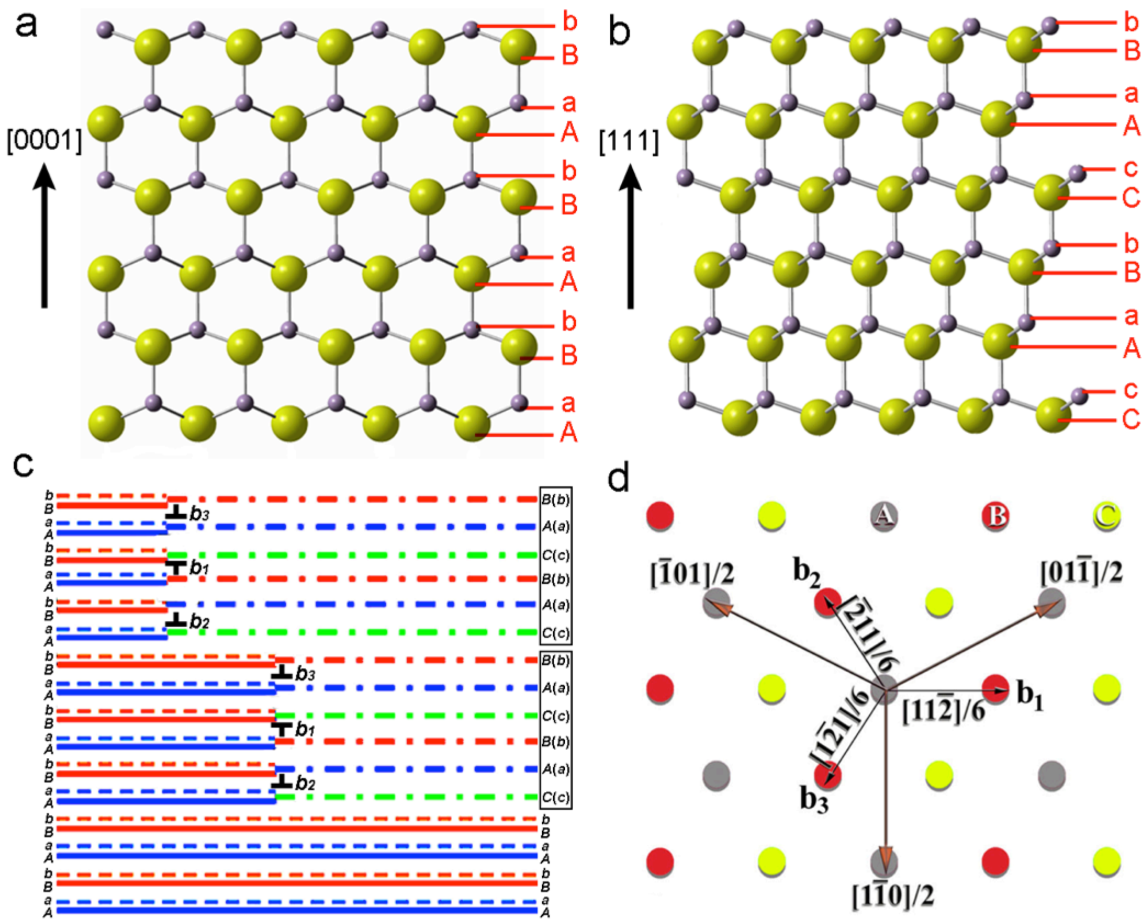

Figure S2. Zero-strain phase transition mechanism. (a) and (b) Atomic structures of wurtzite and zinc blende structures, showing {0001} plane stacking in wurtzite structure with stacking order symbolized as …*AaBbAaBbAaBb*…. and {111} plane stacking in zinc blende structure with stacking order symbolized as …$C(c)A(a)B(b)C(c)A(a)B(b)$…. (c) Schematic illustration of the zero-strain Phase transition mechanism, showing two steps comprising of two sets of dislocations with an order of b_2:b_1:b_3. (d) Three Burgers vectors with the reference of an {111} plane in fcc structure.

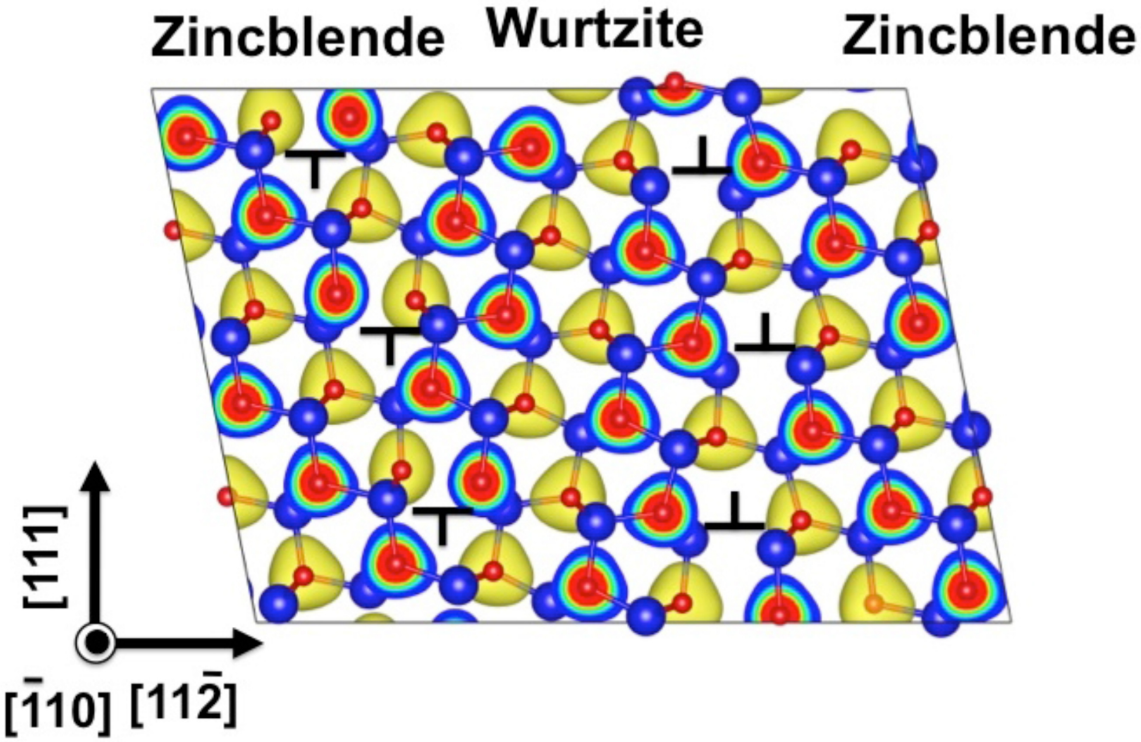

Figure S3. DFT calculation shows atomic structure of a six-layer bi-phase zinc blende AlN and wurtzite AlN in one layer. Three Shockley partials with the Burgers vectors shown in Figure S1 pile up vertically and form the sharp interface which separates the z-AlN from the w-AlN.
